# Personal Exposure Assessment to Wi-Fi Radiofrequency Electromagnetic Fields in Mexican Microenvironments

**DOI:** 10.3390/ijerph18041857

**Published:** 2021-02-14

**Authors:** Raquel Ramirez-Vazquez, Jesus Gonzalez-Rubio, Isabel Escobar, Carmen del Pilar Suarez Rodriguez, Enrique Arribas

**Affiliations:** 1 Applied Physics Department, Faculty of Computer Science Engineering, University of Castilla-La Mancha, Avda. de España s/n, Campus Universitario, 02071 Albacete, Spain; isabelmaria.escobar@uclm.es (I.E.); enrique.arribas@uclm.es (E.A.); 2 Medical Science Department, School of Medicine, University of Castilla-La Mancha, C/Almansa 14, 02071 Albacete, Spain; jesus.gonzalez@uclm.es; 3 Department of Mechanical Engineering, Autonomous University of San Luis Potosi, Academic Coordination of the Huasteca South Region, Tamazunchale, San Luis Potosi 79960, Mexico; pilar.suarez@uaslp.mx

**Keywords:** microenvironments, personal exposure, radiofrequency electromagnetic fields, wi-fi band, risk perception

## Abstract

In recent years, personal exposure to Radiofrequency Electromagnetic Fields (RF-EMF) has substantially increased, and most studies about RF-EMF with volunteers have been developed in Europe. To the best of our knowledge, this is the first study carried out in Mexico with personal exposimeters. The main objective was to measure personal exposure to RF-EMF from Wireless Fidelity or wireless Internet connection (Wi-Fi) frequency bands in Tamazunchale, San Luis Potosi, Mexico, to compare results with maximum levels permitted by international recommendations and to find if there are differences in the microenvironments subject to measurements. The study was conducted with 63 volunteers in different microenvironments: home, workplace, outside, schools, travel, and shopping. The mean minimum values registered were 146.5 μW/m^2^ in travel from the Wi-Fi 2G band and 116.8 μW/m^2^ at home from the Wi-Fi 5G band, and the maximum values registered were 499.7 μW/m^2^ and 264.9 μW/m^2^ at the workplace for the Wi-Fi 2G band and the Wi-Fi 5G band, respectively. In addition, by time period and type of day, minimum values were registered at nighttime, these values being 129.4 μW/m^2^ and 93.9 μW/m^2^, and maximum values were registered in the daytime, these values being 303.1 μW/m^2^ and 168.3 μW/m^2^ for the Wi-Fi 2G and Wi-Fi 5G bands, respectively. In no case, values exceeded limits established by the International Commission on Non-Ionizing Radiation Protection (ICNIRP). Of the study participants (*n* = 63), a subgroup (*n* = 35) answered a survey on risk perception. According to these results, the Tamazunchale (Mexico) population is worried about this situation in comparison with several European cities; however, the risk perception changes when they are informed about the results for the study.

## 1. Introduction

The development and launch of new wireless communication technologies have caused an increase in personal exposure to Radiofrequency Electromagnetic Fields (RF-EMF), and, consequently, social concern has increased about potential adverse effects caused by RF-EMF on human health [1,2,3,4,5,6,7]. 

In this context, the appearance of personal exposimeters significantly improved research possibilities in this field because these devices permit the measurement of RF-EMF intensity in different frequency bands [8,9] in order to be able to compare it with exposure limits defined by national and international standardization organizations [10,11]. Exposimeters are lightweight and portable monitoring devices that discriminate by numerous frequency bands and provide a detailed description of exposure to RF-EMF [12]. Their main advantages are user-friendly handling for participants as volunteers in the studies and the large amount of personal exposure data that can be obtained [13]. 

The aim of conducting studies with exposimeters is to know the personal exposure level to RF-EMF in different microenvironments, such as public transport, outdoor urban areas, other areas inside houses, etc. [4,13,14,15,16,17,18,19,20,21,22,23,24]. However, due to the limitations that these studies may entail, several research projects have also permitted the development of models to estimate exposure by means of sporadic measurements [25,26,27,28,29,30,31,32]. 

Several authors have used different assessment methods, including the characterization of personal exposure based on activities and sources and the assessment of personal exposure with sporadic or long-term measurements [6,8,33,34,35,36]. Other authors have described how to build an exposure activity matrix, both indoors and outdoors [37,38,39]. There are also papers comparing exposure between different zones and different periods of the day [40,41], or describing exposure levels and the contribution of different sources to RF-EMF [3,6,13,42,43,44,45]. 

There are studies monitoring personal exposure to RF-EMF in the daily environment in different types of microenvironments [21,46,47,48,49], and other ones focus on different microenvironments where researchers perform measurements personally [5,18,45,50]. The majority of the studies that compared personal exposure levels to RF-EMF [16,51] with recommended reference levels [52] identified that their results were insignificant and that they were far below these levels; consequently, they complied with this legislation.

The development of emerging technological advances, mainly the increasing use of 4G and 5G networks (fourth and fifth generation, respectively), has led to a significant increase in the installation of antennas that facilitate connection and operation, generating an increase in the discussion about the possible effects of RF-EMF on human health [1,3,6,10,53,54], and this development has prompted researchers to develop personal exposure to RF-EMF studies from this frequency band [55]. Some studies about this field are a summary on the state of investigations on the possible effects of Wi-Fi band networks on public health [56,57], a review that shows seven effects of Wi-Fi bands on animals and human cells [58,59] and the results of a study conducted in primary and secondary schools [60].

When it comes to the 5th Generation (5G) of wireless networks, we refer to new technology in Telecommunication Networks or the deployment of 5G mobile networks; however, when it comes to the Wi-Fi 2G band and the Wi-Fi 5G band, we refer to the 2.4 GHz Wi-Fi band and the 5 GHz Wi-Fi band from 2400–2500 MHz and 5150–5850 MHz frequency bands, respectively. This corresponds to wireless communication systems, and the two currently available Wi-Fi band standards (2.45 GHz and 5 GHz) use electromagnetic fields (EMF) in the radiofrequency (RF) range (according to International Commission on Non-Ionizing Radiation Protection (ICNIRP) guidelines [61]: 100 kHz–300 GHz) for information transmission, with these being the frequency bands where we have measured.

Despite all these studies that have characterized personal exposure to RF-EMF, the population remains concerned about the appearance of diseases with an unknown etiology, hence causing the nocebo effect [62,63].

To review and control maximum allowed exposure levels to RF-EMF, there are international committees such as the International Commission on Non-Ionizing Radiation Protection, the Institute of Electrical and Electronics Engineers (IEEE), the International Committee on Electromagnetic Safety (ICES), the International Electrotechnical Commission (IEC), the American National Standards Institute (ANSI), the World Health Organization (WHO), and the International Agency for Research on Cancer (IARC). These standards are periodically reviewed and updated, considering the new technological implications for exposure to RF-EMF and considering possible effects and risks on health. A revised version of these limits appeared in 2019 for IEEE C95.1-2019 Standard [64], and a new version of the ICNIRP guidelines was published in March 2020 [61]. The ICNIRP establishes that the maximum legal exposure level between 2 and 300 GHz is 50 W/m^2^ for occupational exposure and 10 W/m^2^ for the general public [52,61,65]. Exposure guidelines are not legal documents, but they are recommendations that legislative bodies can use for different purposes.

Wave intensity is measured in W/m^2^; therefore, we may regard this as a power per surface unit. To obtain total power, it would be necessary to integrate the surface subject to study, hence several authors name it power flux density or power density, but in this work, wave intensity and power density are considered as synonyms. 

Most studies on personal exposure to RF-EMF have mainly been developed in Europe. To the best of our knowledge, this is the first study carried out in North America with personal exposimeters, particularly in Mexico, where we know that social concern has been raised about the possible effects of RF-EMF on people’s health. The worry about this electromagnetic wave and the ignorance of exposure levels received in the town of Tamazunchale (San Luis Potosi, Mexico) has awakened an interest to develop this study, reproducing the measurement protocol used by several authors [12,13,21]. An interesting study to be performed in the future would be in the center of Mexico City and in other states in the country. 

To know participants’ opinions about the possible effects of RF-EMF on people’s health and the effect of sharing the results of this measurement, volunteers were asked about their risk perception on this topic through a survey. This survey was applied before the volunteers’ participation and after the volunteers received a detailed report of the results to compare these ones with other studies. This provides information of interest to researchers, because as mentioned before, this is the first study carried out with personal exposimeters and volunteers. In addition, public opinion on this field was unknown, and most people have wondered about the possible effects of these electromagnetic fields on health.

The main objective of this research was to measure personal exposure to RF-EMF from Wi-Fi bands in Tamazunchale, San Luis Potosi, Mexico (96,820 inhabitants, southeast of Mexico City) [66], where we know that national authorities had not approved maximum permitted exposure limits when we carried out the measurement, and where state and local authorities have not defined exposure limits, and no similar studies are known with volunteers in any Mexican town. Likewise, to compare results with another studies in countries where they do have legislation, mainly in Europe, it will be verified whether exposure levels recorded in this study comply with international legislation. In addition to the measurement’s exposure to RF-EMF, risk perception has been assessed by volunteers, to know if the risk perception of people to the potential effect of RF-EMF on health depends on their knowledge about personal exposure levels to RF-EMF.

## 2. Materials and Methods 

### 2.1. Volunteers and Location Ten Second

This experimental study was conducted with 63 volunteers living in the town of Tamazunchale, and they were recruited by an invitation delivered by different means of communication: a local television show, conferences to communicate the research project, personal contacts, and e-mails. Volunteers’ personal data were recorded on an Access Database using Microsoft Access and Google Earth. These data were not public, to respect people’s privacy. 

**Figure 1:**
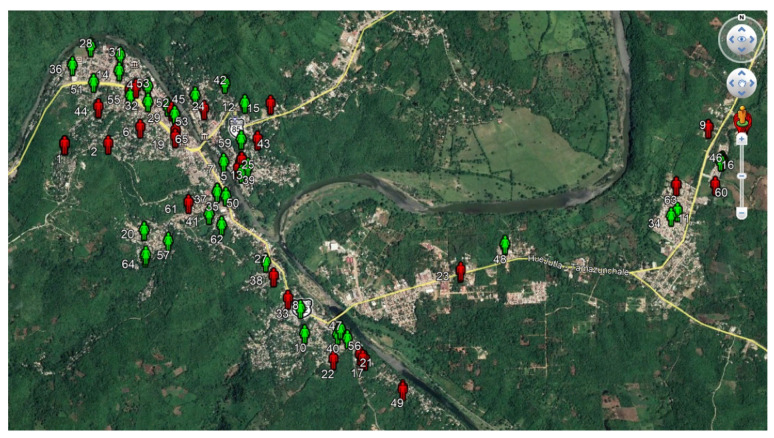
Location of the houses of each participating volunteer (green icon for women and red icon for men).

The location of private houses and workplaces was identified because a member responsible for the measurement process in the research group visited the volunteer to deliver and collect the personal exposimeter. The spatial distribution and location of participants’ houses are shown in Figure 1. 

The municipality of Tamazunchale, San Luis Potosi (Mexico), where this study was conducted, is located between parallels 21°09′ and 21°20′ north latitude; meridians 98°37′ and 98°57′ of west longitude; altitude between 40 and 1400 m; and it occupies 0.6% of the state surface area. It is a mountainous region, where mean annual temperature ranges from 20 to 24 °C, annual precipitation range is 1500–3000 mm and the climate is semi-warm humid with abundant rainfall in summer (51%) and semi-warm humid with rainfall all year round (49%) [67].

### 2.2. Exposimeter and Measurement Protocol 

Measurements were made with three personal exposimeters: Satimo EME SPY 140, properly calibrated by the French company Satimo and configured in the same way before being delivered to volunteers, with the aim of ensuring measurement accuracy concerning time. EME SPY 140 exposimeters are devices measuring 14 frequency bands between 88 MHz and 5.85 GHz (Table 1), and they can record up to 12,540 measurements during periods lasting between 4 and 255 s. The minimum value detected by the exposimeter for each band is: FM (Frequency Modulation): 6.631 µW/m^2^; TETRA (Terrestrial Trunked Radio, TV4&5 (Television): 0.265 µW/m^2^; GSM (Global System for Mobile Communications), DCS (Digital Cordless Systems), DECT (Digital Enhanced Cordless Telecommunications), UMTS (Universal Mobile Telecommunications System), Wi-Fi 2G (Wireless Fidelity) band: 0.066 µW/m^2^; and TV3, WiMAX (Worldwide Interoperability for Microwave Access), Wi-Fi 5G band: 1.061 µW/m^2^. Out of the 14 frequency bands measured by the exposimeter (Table 1), only the Wi-Fi 2G band (2400–2500 MHz) and the Wi-Fi 5G band (5150–5850 MHz) were taken into consideration.

Technical difficulties (effects of the human body, field strength, and polarization rapidly varied over time-fading, calibrating equipment, etc.), methodological problems (measuring protocol) and data analysis-type drawbacks (non-detects, using means, medians, etc.) [14,68,69] must be taken into account to prevent conditioning results for the research [13,15,43,70,71,72,73], as mentioned in the next section.

The exposimeter was configured to measure every 10 s for 25 h, among which only 24 h were considered; half an hour before and after the measurement process was discarded to avoid potential errors. 

The measurement protocol used to perform measurements of personal exposure to RF-EMF allowed us to select and instruct participants as volunteers in the study, as well as to provide processing and data analysis [12]. An exposimeter was handed to each volunteer at home. A research team member visited each volunteer to deliver the exposimeter, to explain the measurement protocol in detail, and to indicate precautions they should consider during the measurement process. Participants received and signed the measurement protocol and the personal data protection policy form; they received a personal diary in which they recorded entry and exit time for each visited microenvironment, using a plastic wristwatch provided to them.

Volunteers had to live a normal life, avoiding the use of their mobile phones as much as possible. In the case that volunteers carried their phones, they were placed on the opposite side of the exposimeter or turned off. Volunteers were informed that, during the measurement process or at least when carrying the exposimeter (inside a bag hanging on their back), it was not possible to make calls, but they may receive calls and should register them in the diary (starting and finishing time) because this measurement was identified and deleted in the data processing and data analysis. 

They were also told not to use Wi-Fi bands from their mobile phone or laptop during the measurement time. Obviously, we trusted that they followed our indications, although they were asked several questions to check that our requirements were met. The volunteer was not allowed to be connected to a Wi-Fi band network. In case that the contrary happened for any emergency, it had to be registered in the personal diary where time and place from start to finish were recorded, with the same objective, to remove measurements registered during this period.

Volunteers always carried their personal exposimeter in a plastic backpack hung on their body so that its transportation would be more comfortable while they carried out their daily activities; for example, going to work, walking, strolling, shopping. When volunteers were still, they had to leave the exposimeter near them, but far from walls and never on the floor or near electronic devices; therefore, when they were sleeping, they had to leave the exposimeter near them. Neither the wristwatch nor the backpack interfered with measurements taken by the exposimeter. Once the measurement period concluded, the researcher in charge visited volunteers to collect the equipment and material supplied to them and downloaded the data to verify that measurement records were correct. 

When the exposimeter made a mistake and did not record measurements of the 25-h period, the process was repeated if the volunteer agreed. This happened three times—with one volunteer the device registered only 12 h and the volunteer took part again, repeating measurements and not considering the wrong ones; however, two volunteers were rejected during the statistical analysis because one volunteer did not have complete records for the 24 h measurements and the other volunteer did not have a complete personal diary, but they did not participate again in the measurement process so the measurements were not taken into consideration because of this reason. Subsequently, measurements for each volunteer were processed and analyzed by the researcher, then, the report corresponding to these measurements was prepared and delivered to each volunteer for their information, mainly by mail.

The variable selected to study personal exposure was wave intensity; although several authors name it power flux density, we believe that the term intensity is more accurate, expressed in W/m^2^. Most of the performed studies have used different units and submultiple units of this measurement such as µW/cm^2^ and mW/m^2^, including electric field units (V/m). However, we are convinced that it is better to use wave intensity. Nevertheless, since device values are extremely sensitive, we have used units in the region of µW/m^2^, hence values are easy to process and represent, and they vary between 0 and 1000 most times. Typical units used for personal exposimeters are W/m^2^. 

Mean personal exposure levels were calculated (measured every ten seconds) for the Wi-Fi 2G band (2400–2500 MHz) and the Wi-Fi 5G band (5150–5850 MHz), later they were classified by six microenvironments: home, workplace, outside, schools, travel, and shopping, to determine exposure in the microenvironments where volunteers were present. In addition, exposure levels received at every volunteer’s home were calculated, this being the microenvironment where they spent most of the time, and finally, they were represented through a georeferenced intensity map with such values.

### 2.3. Microenvironments and Statistical Analysis

A register of all locations that volunteers visited as well as the entry and exit time for each one was recorded in each volunteer’s diary. This information was very useful to classify the measurements recorded in the 15 microenvironments that we initially considered: home; outside; workplace; friend and family place; car; public transport; restaurant, bar, café, disco, etc.; sport hall; cinema, theater, concert, etc.; university; school, kindergarten; hospital; other building; shopping; outside the city. However, since volunteers spent most of their time in the following microenvironments: home, workplace, outside, schools, travel, and shopping, measurements were classified and considered for their respective analysis only in the latter 6 microenvironments.

Once these measurement classifications were made in different microenvironments, the statistical data analysis was carried out using the exposimeter software EME SPY Analysis V3.20 from TEMSYSTEM (Madrid, Spain), R Studio software version 3.5.1 (Boston, MA, USA) and IBM SPSS Statistics software version 22 (Statistical Package for the Social Sciences, IBM, Armonk, NY, USA). These calculations were made with the electromagnetic wave intensity values, expressed in μW/m^2^, as mentioned above.

Before statistical analysis, all records were revised to identify where errors had been detected, and they were deleted. Likewise, there was a search for values registered below the detection limit of the exposimeter in each frequency band. Values registered as non-detects received one treatment, and they were replaced by the detection limit divided by two [69]. The non-detects data percentage is between 80 and 85% in these two bands.

### 2.4. Volunteers and Measurements

Measurements were carried out between 2017 and 2018, considering a measurement period of 24 h. A total of 65 volunteers participated in the study, but only 63 of them were considered to provide valid measurements. As we mentioned before, two volunteers were rejected during the statistical analysis due to the following errors: one volunteer did not have complete records for the 24 h measurements and the other one did not have a complete personal diary where time and visited microenvironments were recorded. A total of 43% of the participants were male and 57% were female. 

The exposimeters measured a total of 1512 h and the records of each measurement were in 10 s intervals, hence a total of 544,320 registers were obtained. Measurements were registered from 14 frequency bands subject to study, representing 7,620,480 data units; however, in this work, only intensity levels from the Wi-Fi 2G band (2400–2500 MHz) and the Wi-Fi 5G band (5150–5850 MHz) were considered for each microenvironment. These are the frequency bands measured by the exposimeter both in Europe and Mexico, as explained in the introduction, and thus we could compare our results with those of other studies.

Finally, due to the volunteers spending more time at home, a geostatistical analysis was performed using the Kriging interpolation method with ArcGIS software to represent levels recorded at home through an intensity map, and to identify the city areas with greater intensity, that is to say, spot measurement.

### 2.5. Geostatistical Analysis

Kriging is an advanced geostatistical method that generates an estimated surface from a scattered set of points with different values of a physical magnitude. Using the Kriging tool involves an interactive investigation of the spatial behavior of the phenomenon represented by the values before you select the best estimation method for generating the output surface.

This method uses a weighted mean of the available data, with the weights depending not solely on the distance but also on the geometry of samples’ location, considering the structure of the spatial correlation deduced from the analysis of the variograms, that is to say, the autocorrelation of the values of a variable taking into consideration its geographic location, and the result of kriging is a map including interpolated values of the variable [74,75]. ArcGIS version 10.6 software provided by ESRI Spain (Madrid, Spain) was used for interpolation analysis.

### 2.6. Identification of Risk Perception

In order to know risk perception from the volunteers’ study about the possible effects of exposure to RF-EMF on human health, participants were asked to answer a survey elaborated with Google Forms and sent by email. This survey was applied before the volunteers’ participation and after the volunteers received a detailed report of their measurements registered during the measurement process. The aim was to identify whether their risk perception changed once they have information about measurements and personal exposure levels to RF-EMF in the different microenvironments visited when carrying the exposimeter.

Risk perception was identified by the study volunteers before and after the study through a survey. In the survey, they answered according to their perception: from 1 to 5, where 1 is Not Dangerous, 2 is Not Very Dangerous, 3 is A Little Dangerous, 4 is Quite Dangerous and 5 is Very Dangerous.

The survey was answered by 36 people, although 35 of them were taken as valid because one of them was incomplete. The statistical analysis was conducted with IBM SPSS V22 from the results obtained in the survey. A t-test for two related or paired samples was used, investigating possible significant differences between the risk perception before and after the study, and *p* < 0.05 was considered statistically significant. 

#### Our Hypotheses Were

**Hypotheses** **(H_0_):** *Risk perception before the study is equal to the risk perception after the study and there are no significant differences between both results; therefore, knowledge or ignorance of personal exposure to RF-EMF does not affect or modify their perception.*

**Hypotheses** **(H_1_):** *Risk perception before the study is different to the risk perception after the study and there are significant differences between both results; therefore, knowledge of personal exposure to RF-EMF modifies their perception.*

The results were compared with those of risk perception by European volunteers in order to know whether there were differences or not, and to identify if the risk perception of people of the potential effect of RF-EMF on health depends on their knowledge about RF-EMF personal exposure levels.

## 3. Results

### 3.1. Temporal Characterization of Personal Exposure to RF-EMF from Wi-Fi Bands

**Figure 2:**
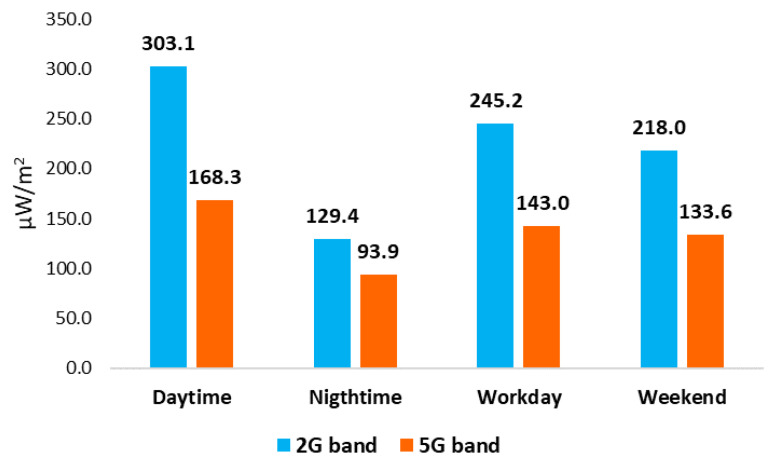
Registered mean for Radiofrequency Electromagnetic Fields (RF-EMF) from Wi-Fi bands by time period and type of day (μW/m^2^).

In Figure 2 and Table 2, we can see the registered mean for RF-EMF from the Wi-Fi 2G band (2400–2500 MHz) and the Wi-Fi 5G band (5150–5850 MHz) by time period and type of day. We can see that minimum values were registered at nighttime, with 129.4 μW/m^2^ and 93.9 μW/m^2^, and maximum values were registered at daytime, with 303.1 μW/m^2^ and 168.3 μW/m^2^ for the Wi-Fi 2G band and the Wi-Fi 5G band, respectively.

### 3.2. Spatial Characterization of Personal Exposure to RF-EMF from Wi-Fi Bands

**Figure 3:**
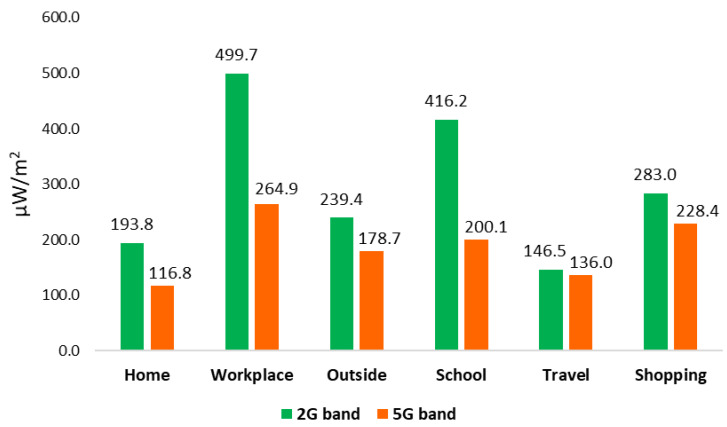
Registered mean for RF-EMF from Wi-Fi bands in each microenvironment (μW/m^2^).

In Figure 3 and Table 3, we can see the registered mean for RF-EMF from the Wi-Fi 2G band and the Wi-Fi 5G band in each microenvironment. The mean minimum values registered were 146.5 μW/m^2^ in travel from the Wi-Fi 2G band and 116.8 μW/m^2^ at home from the Wi-Fi 5G band, and the maximum values registered were 499.7 μW/m^2^ and 264.9 μW/m^2^ in the workplace for the Wi-Fi 2G and Wi-Fi 5G bands, respectively.

In Table 2 and Table 3, we can see the great variability for the data; the standard deviation is much higher than the mean, which means that the data oscillate between the minimum and the maximum limits numerous times throughout our measurements. The explanation is that the Wi-Fi band works on demand, that is, if no one requests any service there is no signal—it is only activated when someone requires some information from the Internet.

Table 2 shows mean values registered in the Wi-Fi 2G and Wi-Fi 5G bands by measured time period and type of day. The main source contributing to a greater extent to the recorded personal exposure was daytime, both for the Wi-Fi 2G band and the Wi-Fi 5G band, and the lowest was nighttime, also in both the Wi-Fi 2G and Wi-Fi 5G bands. When revising and analyzing mean values registered from the Wi-Fi 2G and Wi-Fi 5G bands of each of the six microenvironments, we observe that the main source contributing to a greater extent to the recorded personal exposure was at the workplace, both for the Wi-Fi 2G and Wi-Fi 5G bands, and the lower extent was in travel for the Wi-Fi 2G band and at home for the Wi-Fi 5G band, respectively (Table 3).

### 3.3. Spot Measurements of Personal Exposure to RF-EMF from Wi-Fi Bands 

Volunteers spent more time at home; therefore, most of the time they were exposed to RF-EMF from their Wi-Fi band at home and the Wi-Fi band from their neighbors because they are always connected to a Wi-Fi band network and they all have their Wi-Fi connection at home or one of their neighbors.

**Figure 4:**
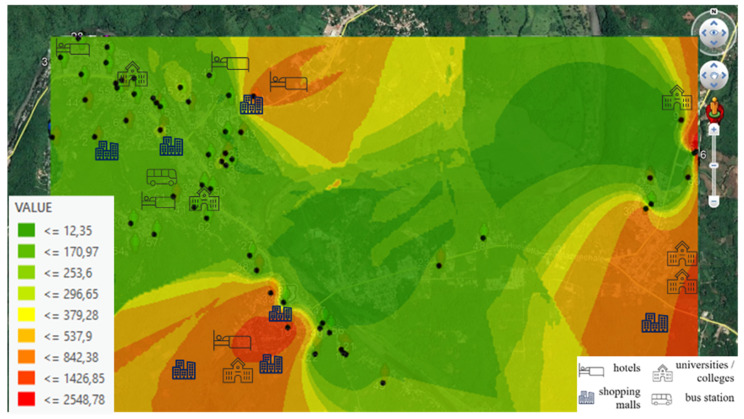
Georeferenced Map with the Wi-Fi 2G band (2400–2500 MHz) exposure levels (μW/m^2^), recorded at volunteers’ homes indicated with black dots.

**Figure 5:**
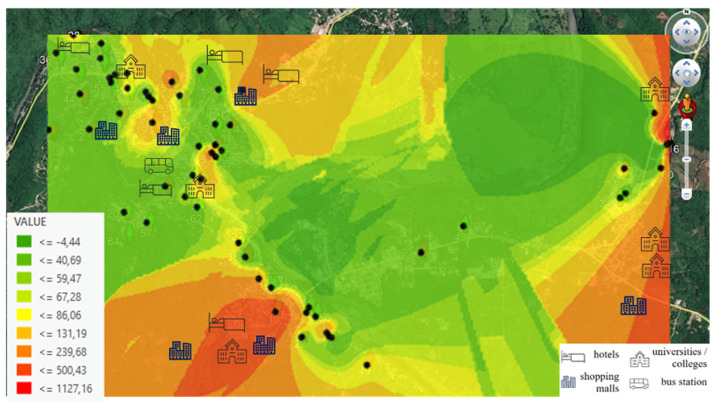
Georeferenced Map with the Wi-Fi 5G band (5150–5850 MHz) exposure levels (μW/m^2^), recorded at volunteers’ homes indicated with black dots.

In Figure 4 and Figure 5, we can see intensity levels for RF-EMF recorded at each volunteer’s home (μW/m^2^). This map allows us to view areas with a greater intensity. Figure 4 shows intensity levels from the Wi-Fi 2G band (2400–2500 MHz) and Figure 5 shows intensity levels from the Wi-Fi 5G band (5150–5850 MHz).

The highest level is shown in the red shaded points, and the explanation is that these houses are surrounded by Wi-Fi bands coming from hotels, universities/colleges, bus stations (in the case of the town center), and above all, and Wi-Fi band from local shops. In both figures, we can see places where Wi-Fi band connections affect exposure levels to be recorded at each volunteer’s home. In addition, if we compare Figure 4 and Figure 5, we observe that the Wi-Fi 5G band (5150–5850 MHz) is more present in local shops, as in the case of the town center. 

### 3.4. Identification of Risk Perception about RF-EMF on Health 

**Figure 6:**
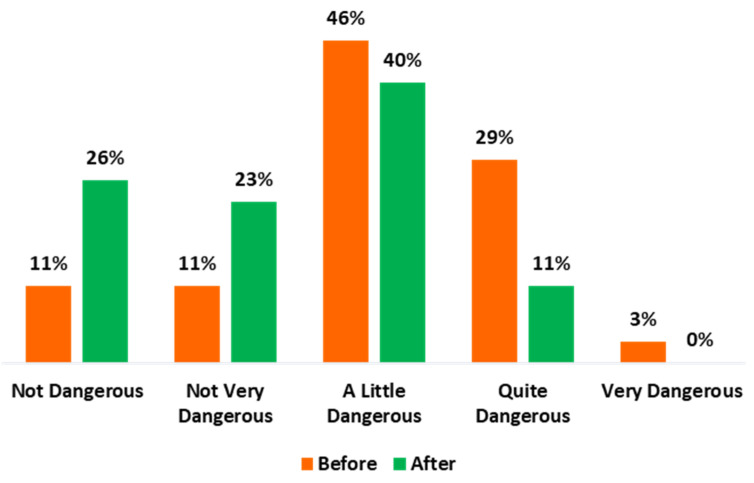
Risk perception by volunteers about possible effects of RF-EMF on people’s health.

In addition to measurements of personal exposure to RF-EMF, risk perception was identified by study volunteers about potential RF-EMF effects on health. Volunteers answered the survey from 1 to 5, where 1 is Not Dangerous, 2 is Not Very Dangerous, 3 is A Little Dangerous, 4 is Quite Dangerous and 5 is Very Dangerous. The results show that before their participation, 11% of the volunteers considered RF-EMF to be not dangerous, another 11% considered it to be not very dangerous, 46% considered it a little dangerous, 29% quite dangerous and only 3% considered it to be very dangerous; however, their risk perception changed after receiving results for the study, as shown in Figure 6, and 0% considered it to be very dangerous.

**Figure 7:**
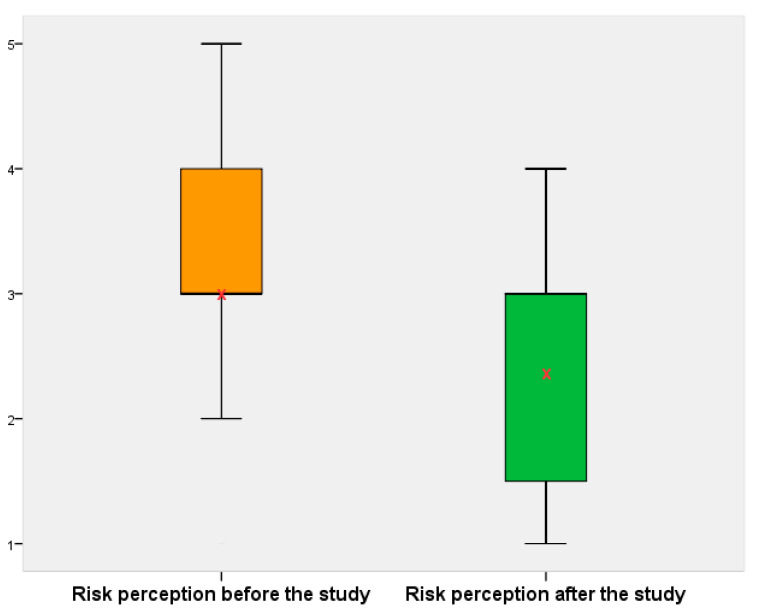
Comparison of risk perception before and after the study. The arithmetic mean of each group is depicted with a red “✕”

When comparing results for risk perception before and after the volunteer was provided by the researcher with a report about the exposure levels, and an explanation about these results, their perception changed (Figure 6 and Figure 7). That is, before participating in the survey and receiving information, they were more concerned, and it is highlighted that the lack of information on this field produces concern and uncertainty in the population. Thus, risk perception about exposure to RF-EMF depends on their knowledge in this field. 

The statistical analysis results obtained for risk perception before are: mean = 3, median = 3 and mode = 3, the results for risk perception after are: mean = 2.4, median = 3 and mode = 3, and the t-test result is *p*-value < 0.01 (*p* < 0.05 was considered statistically significant); therefore, we have statistically significant differences to reject H_0_ and accept H_1_, that is, the risk perception before the study is different to the risk perception after the study and there are significant differences between both results; therefore, knowledge of personal exposure to RF-EMF modifies their perception.

Regarding the recorded results of personal exposure to RF-EMF, there are no differences in exposure between volunteers that had negative perceptions compared to those who did not.

## 4. Discussion

Most measurement studies on personal exposure to RF-EMF have been developed in Europe; to the best of our knowledge, there is no study conducted in Mexico. The measurement protocol used to carry out this project is similar to the protocol applied in some studies [12,13,21,76] since it provides basic patterns to select and instruct participants in the study during the measurement process, as well as to manage and analyze data. Measurements were carried out for 25 h, recording measurements every 10 s. However, only 24 h were considered since measurements recorded during the first half an hour and those registered during the last half an hour were deleted, and only exposure to RF-EMF measurements received by volunteers during their daily activity were included. Volunteers carried the personal exposimeter hanging on their body and their mobile phone on the opposite side of the equipment, preventing its use. Even so, measurements registered during some calls were deleted to minimize interferences in the results.

If we compare minimum and maximum levels recorded by microenvironment, we can see that, on the one hand, the minimum values registered were 146.5 μW/m^2^ in travel from the Wi-Fi 2G band (2400–2500 MHz) and 116.8 μW/m^2^ at home from the Wi-Fi 5G band (5150–5850 MHz), and the maximum values registered were 499.7 μW/m^2^ and 264.9 μW/m^2^ at the workplace for the Wi-Fi 2G and Wi-Fi 5G bands, respectively. On the other hand, if we compare minimum and maximum levels by time period and type of day, we can see that minimum values were registered at nighttime, with 129.4 μW/m^2^ and 93.9 μW/m^2^, and maximum values were registered at daytime, with 303.1 μW/m^2^ and 168.3 μW/m^2^ for the Wi-Fi 2G and Wi-Fi 5G bands, respectively.

When we compared these values with the results of Birks obtained in Denmark, the Netherlands, Slovenia, Switzerland, and five towns in Spain (Gipuzkoa, Granada, Menorca, Sabadell, and Valencia), we identified that the minimum value from Wi-Fi bands was 0.1 μW/m^2^ and the maximum value was 49.2 μW/m^2^ [1]. Therefore, we observe that our values were higher, and this may be because measured microenvironments are urban areas where public institutions and local shops are located, then contributing to intensity levels suffered by volunteers at home, as shown on intensity maps.

In the same way, if we compare the results for these measurements performed in a Mexican city with similar measurements performed in a Spanish city, we identify that the minimum value from Wi-Fi bands was 3.9 μW/m^2^ and the maximum value was 86.9 μW/m^2^ [21]. We still observe that the results of this work remain higher, but if we compare the results of this work with those obtained in the laboratory measurements by Khalid in Oxfordshire, we identify that the maximum time-averaged power density from a laptop would be 220 μW/m^2^ at a distance of 0.5 m [60], a similar value to results herein presented. Although we are comparing our measurements with the study conducted by Khalid because they have measured across the Wi-Fi band, we observe that this experiment was carried out under different conditions from ours.

A study recently performed in Spain by Fernández alludes to a maximum Wi-Fi band exposure of 441 μW/m^2^ within a laboratory—a value that is comparable to our measurements [77]. Finally, we observed similar values, even higher, in another study carried out in Belgium. They registered 53.05 μW/m^2^ at schools and 13,581.19 μW/m^2^ at home and public places [78]—this last value is −29 dB from the maximum allowed by the ICNIRP. Our highest values are at −43 dB.

In a recently published study, we carried out measurements in a Jordanian University area [50] and we obtained comparable values in the Wi-Fi bands. At one point, we recorded a maximum value of 385 μW/m^2^, and now we have recorded a value of 500 μW/m^2^ in Mexico. As stated before, there is no approved national legislation with exposure levels in Mexico. In addition, this type of measurement has never been carried out, and therefore, we cannot compare our data with another study under similar conditions.

In the studies carried out by our research group and the ones conducted by Verloock, wherein the Wi-Fi band is subject to measurements, the following mean values (minimum and maximum) have been obtained (see Table 4).

**Figure 8:**
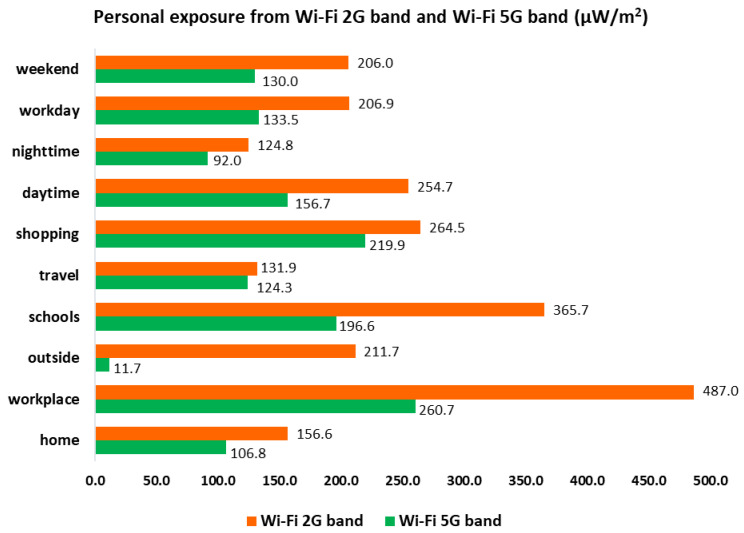
Mean exposure from the Wi-Fi 2G and Wi-Fi 5G bands by microenvironments, time period and type of day (μW/m^2^).

As observed in light of these results, there are high values registered—a reason why we have deeply revised measurements registered in every single microenvironment, focusing on the higher measurements. Out of the 63 volunteers, we have focused our attention on those with higher measurements. In total, we have reanalyzed 20 volunteers and observed the following:

- At night, from 8 p.m. to 7 a.m. (approximately), there is a signal in both Wi-Fi bands (35% in one and 65% in the other) that is intense, reaching almost 67,000 μW/m^2^, which is a high value but not exceeding ICNIRP limits. This signal lasts only for very few seconds but occurs about four times during the night. When calculating the mean, this high value increases the mean for the microenvironment as the signal is so high but lasts for a very short amount of time, and its contribution to the mean is not as relevant as expected. If we exclude the maximum values registered, we obtain Figure 8.
- In the “house” microenvironment, the mean in the Wi-Fi 5G band changes from 193.8 μW/m^2^ to 156.6 μW/m^2^, so it decreases by 20%, although it is still a high value. If we compare these values with Figure 2 and Figure 3, we observe that there is little difference because these maximum values are rarely registered.
- We do not exactly know the reason for this high signal registered at isolated moments, but we suppose that it is related to police operations. For obvious reasons, we cannot obtain further information about this fact.

Legislation stipulating maximum permitted limits applicable to Mexico is based on the International Commission on Non-Ionizing Radiation Protection [52]. When we carried out the measurement, there was no national legislation approved and published; however, the Federal Institute of Telecommunications (IFT) of Mexico had informed us about its publication from December 2016 to February 2017 of the preliminary draft of technical provision IFT-007-2016 [80]. This was subject to a public consultation and we expected the publication at the end of 2019 or 2020 of the Technical Provision IFT-007-2019, which establishes the maximum human exposure limits to Non-Ionizing Radiofrequency Electromagnetic Radiation in the range from 100 kHz to 300 GHz in the vicinity of radio communications stations, published in 2020 [81]. State and local authorities have not defined exposure limits. If we compare our results with limits stipulated by the ICNIRP [52,61] and IFT-007-2019 [81], we can conclude that they are far below the limits.

The Global System for Mobile Communications is the most popular standard system for cell phones in the world. However, a difference that we may identify when comparing frequency bands used in Europe and Latin America, particularly in Mexico, is that GSM 900/GSM 1800 MHz is the band used in Europe, and GSM 850/GSM 1900 MHz is the one used in most Latin American countries, in particular in Mexico [82]. However, in the case of Wi-Fi bands, frequencies are similar to the Wi-Fi 2G band and the Wi-Fi 5G band for Europe and Mexico, and values were found to be between 2400–2500 MHz and 5150–5850 MHz, respectively [83], frequencies at which EME SPY 140 measures.

An important aspect of this sort of work cannot be carried out without the inestimable volunteers’ collaboration, and this is the reason why a small sample of the population was selected. The orography of the town and the lifestyle of the Tamazunchale population have demanded us to solve some small inconveniences, but we believe this has permitted us to obtain a Wi-Fi band intensity map in a medium-sized town. In addition to difficulties when contacting participants and performing measurements, we have found differences between frequency bands operating in Mexico and frequency bands measured by the exposimeter in use, and this is the reason why we decided to focus the study on the Wi-Fi 2G band (2400–2500 MHz) and the Wi-Fi 5G band (5150–5850 MHz). This is one of the difficulties relevant to us because for future studies, we intend to use new exposimeters, since these exposimeters must measure all frequencies that operate in the place where the study is conducted. This may be an interesting suggestion for companies manufacturing these devices, offering exposimeters operating in all frequency bands available all over the world and facilitating work to researchers in this field.

On risk perception, if we compare the results of the risk perception by Mexican volunteers participating in this study with the results of the risk perception by European volunteers [21,76], we can know that, in this case, the Tamazunchale (Mexico) population is worried about this situation in comparison with several European cities.

We would like to highlight that risk perception changed (decreased) when participants received information about measurements recorded during their participation in the study. Risk perception before the study is different to risk perception after the study, and our t-test result is *p* < 0.01 (*p* < 0.05 was considered statistically significant). There are statistically significant differences between both results; therefore, knowledge of personal exposure to RF-EMF modifies their perception. However, there are no differences in personal exposure to RF-EMF between volunteers that had negative perceptions compared to those who did not.

The mean value for data in Figure 7 is 3.0, in this case, coinciding with mode and median values. If we compare these values with results from the study on risk perception carried out in Spain [21], we observe that these values are different—the mean is 2.2, and the mode and median are 2.0, on a scale from 1 to 5. The study carried out in Australia [76] provided a mean (SD) value (from 1 to 7) for risk perception to RF-EMF from mobile phone base stations of 4.02 (1.67) for basic information, 3.82 (1.62) for precaution messages, and 3.97 (1.72) for personal exposure measurement groups. The personal exposure measurement group was more confident, and they could protect themselves from RF-EMF to a greater extent than the precaution or basic information groups. Making this comparison, we can deduce that people are more worried in Mexico than in Europe, and we believe that one of the factors influencing this fact is the lack of information and knowledge about personal exposure levels to RF-EMF; therefore, the greater the knowledge about RF-EMF, the lower the risk perception associated with these fields.

We have compared our results with two studies [21,76] because they have performed measurements for personal exposure to RF-EMF, provided RF-EMF information and shared results with volunteers. In these studies, they have also evaluated risk perception.

Other studies also refer to risk perception, but the results were only obtained from questionnaire application without carrying out measurements of personal exposure [84,85,86,87,88,89,90,91], and other studies that measured exposure to RF-EMF did not evaluate risk perception [15,79,92,93]. As stated in the introduction, most studies on this topic are conducted in Europe, not in Mexico, and therefore this is a novel study.

## 5. Conclusions

When comparing personal exposure levels to Wi-Fi band Radiofrequency Electromagnetic Fields registered in this study with maximum limits allowed by the ICNIRP and IFT-007-2019, we observed that these levels are far below the levels established for the general public. The main source of exposure was the Wi-Fi 2G band, which is the most used in this town. The absence of state and local legislation does not suppose an increase in exposure levels, or the infringement of maximum levels permitted by international regulations. According to the results of risk perception surveys, the Tamazunchale (Mexico) population is worried about this situation in comparison with several European population, but perception changes when they are informed about the results of the study.

In the near future, when the Wi-Fi 5G band is already implemented in Mexico, we intend to repeat the measurements in order to know whether there is any difference and to ensure that international suggestions are met. In addition, we intend to make use of new personal exposimeters to include all frequency bands and compare current results with the ones obtained in Europe. As this is a pioneer study, one of the main problems we faced was the difference between some frequency bands measured by the device and the operating ones in Mexico. This is the reason why results solely from Wi-Fi bands were obtained and presented. This is a very important aspect to be considered for future research since not all devices are homogeneous regarding frequency bands measured and the ones operating all over the world.

We would like to encourage other researchers to work on this field of Applied Physics because there are many studies in Europe but not so many in Latin America (North and South), and we believe it is very important to have information about the ocean of radiofrequency waves surrounding us, ensuring that international, state, and local levels are respected (if such limits exist).

## Figures and Tables

**Table 1:** Measured frequency bands for EME SPY 140 exposimeter.

Band	Description of Frequency Bands	Frequency (MHz)
FM	Radio broadcast transmitter	88–108
TV3	Television broadcast transmitter	174–223
TETRA	Mobile communication for closed groups	380–390
TV4&5	Television broadcast transmitter	470–830
GSM uplink	Transmission from handset to base station	880–915
GSM downlink	Transmission from base station to handset	925–960
DCS uplink	Transmission from handset to base station	1710–1785
DCS downlink	Transmission from base station to handset	1805–1880
DECT	Digital Enhanced Cordless Telecommunications	1880–1900
UMTS uplink	Transmission from handset to base station	1920–1980
UMTS downlink	Transmission from base station to handset	2110–2170
Wi-Fi 2G	Wireless local area network	2400–2500
WiMAX	Worldwide interoperability for microwave access	3400–3800
Wi-Fi 5G	Wireless local area network	5150–5850

**Table 2:** Mean of personal exposure to RF-EMF by time period and type of day (μW/m^2^).

	Wi-Fi 2G Band				Wi-Fi 5G Band			
	Daytime	Nighttime	Workday	Weekend	Daytime	Nighttime	Workday	Weekend
Min	0	0	0	0	0.05	0.05	0.05	0.53
Mean	303.1	129.4	245.2	218.0	168.3	93.9	143.0	133.6
Mode	0.1	0.1	0.1	0.1	0.5	0.5	0.5	0.5
SD	2400	1400	2100	1700	1600	1200	1500	1300
P5	0.1	0.1	0.1	0.1	0.5	0.5	0.5	0.5
P50	0.45	0.07	0.27	0.1	0.53	0.53	0.53	0.53
P90	315.7	55.8	215.5	61.3	73.1	28.1	72.2	15.7
P95	1090	230.8	776.3	566.2	401.4	200.6	367.1	112.6
P99	5650	2820	4425	5500	3360	1430	2470	3350
Max	66,300	66,300	66,300	66,300	66,300	66,300	66,300	66,300

**Table 3:** Mean of personal exposure to RF-EMF by microenvironment (μW/m^2^).

	Wi-Fi 2G Band						Wi-Fi 5G Band					
	Home	Workplace	Outside	School	Travel	Shopping	Home	Workplace	Outside	School	Travel	Shopping
Min	0	0	0	0	0	0.01	0.05	0.53	0.05	0.05	0.05	0.53
Mean	193.8	499.7	239.4	416.2	146.5	283.0	116.8	264.9	178.7	200.1	136.0	228.4
Mode	0.1	0.1	0.1	0.1	0.1	0.1	0.5	0.5	0.5	0.5	0.5	0.5
SD	2000	2300	2300	2300	1500	1600	1500	1500	1800	1700	1400	1300
P5	0.1	0.1	0.1	0.1	0.1	0.1	0.5	0.5	0.5	0.5	0.5	0.5
P50	0.13	2.08	0.07	2.55	0.17	0.45	0.53	0.53	0.53	0.53	0.53	0.53
P90	98.8	1292.3	98.1	722.8	71.3	155.5	32.1	371.02	49.1	156.6	61.3	104.7
P95	397.3	2279	324.9	1630	409.7	1738	218.5	1296	164.1	554.0	351.4	590.9
P99	3970	7870	6530	6940	3230	7490	1710	4890	41,640	3930	2440	7590
Max	66,300	66,300	66,300	66,300	66,300	66,300	66,300	66,300	66,300	66,300	66,300	21,200

**Table 4:** Minimum and maximum values obtained in some measurement studies from Wi-Fi bands (μW/m^2^).

Publication	Location	Minimum Value	Maximum Value
Ramirez-Vazquez, et al., 2019 [21]	Albacete (with volunteers)	3.9 (Wi-Fi band)	86.9 (Wi-Fi band)
Ramirez-Vazquez, et al., 2019 [45]	Albacete (mobile phone base stations during temporary events)	29.0 (DCS-DL band)	1114 (GSM-DL band)
Ramirez-Vazquez, et al., 2020 [79]	Albacete (school building: inside and outside)	0.61 (Wi-Fi band)	121 (Wi-Fi band)
Ramirez-Vazquez, et al., 2020 [50]	Jordan (University area)	1.41 (Wi-Fi band)	385 (Wi-Fi band)
Verloock et al., 2014 [78]	Belgium (Public places)	53.05 (Wi-Fi band)	13,580 (Wi-Fi band)
This paper	Mexico (with volunteers)	93.3 (Wi-Fi band)	500 (Wi-Fi band)

## Data Availability

Not applicable.
